# Storage on Maternal Plants Affects Temperature Requirements during Germination in *Rumex obtusifolius*

**DOI:** 10.3390/plants12132403

**Published:** 2023-06-21

**Authors:** Arvind Bhatt, Xingxing Chen, David J. Gallacher, Shyam S. Phartyal, Luis Alfonso Rodriguez-Paez, Yirlis Yadeth Pineda-Rodriguez, Marcelo F. Pompelli, Aftab Jamal, Roberto Mancinelli, Emanuele Radicetti

**Affiliations:** 1 Lushan Botanical Garden, Chinese Academy of Sciences, Jiujiang 332900, China; chengxg@gmail.com; 2 Northern Hub, Charles Darwin University, Casuarina, NT 0909, Australia; david.gallacher@cdu.edu.au; 3 School of Ecology and Environment Studies, Nalanda University, Rajgir 803116, India; shyamphartyal@gmail.com; 4 Facultad de Ciencias Agrícolas, Universidad de Córdoba, Montería 230002, Córdoba, Colombia; larguez@fca.edu.co (L.A.R.-P.); yadeth@fca.edu.co (Y.Y.P.-R.); marcelo@fca.edu.co (M.F.P.); 5 Department of Soil and Environmental Sciences, Faculty of Crop Production Sciences, The University of Agriculture, Peshawar 25130, Pakistan; aftabses98@gmail.com; 6 Department of Agricultural and Forestry Sciences (DAFNE), University of Tuscia, 01100 Viterbo, Italy; 7 Department of Chemical, Pharmaceutical and Agricultural Sciences (DOCPAS), University of Ferrara, 44121 Ferrara, Italy; emanuele.radicetti@unife.it

**Keywords:** aerial seed bank, light, seed germination, temperature, seed respiration

## Abstract

Aerial seed banks facilitate population persistence by extending the temporal range of seed dispersal. Knowing the temporal range of germination will improve our understanding of the relationship between seed germination dynamics and aerial seed bank storage duration. We tested the effects of temperature (12/12 h of 5/10, 10/20, 20/30 and 25/35 °C) and light variation (12 h light/12 h darkness and 24 h darkness per day) on germination of *Rumex obtusifolius* L. seeds retained in an aerial seed bank for 0, 2, 4, 6, 8 and 10 months. Freshly harvested *R. obtusifolius* were non-dormant and exhibited germination rates of up to 92%. Overall, seeds of *R. obtusifolius* germinated reliably at all but the lowest temperature (5/10 °C). Seeds maintained high viability throughout the collection period, indicating that fluctuating weather conditions had little influence on seed germination. Thus, the species can maintain viable seeds in aerial storage for up to 10 months and contribute viable seeds to the soil seed bank year-round. This ability to maintain a renewed soil seed bank contributes to the species’ strong resilience in colonizing disturbed areas and makes it a difficult weed to control.

## 1. Introduction

Asynchronous seed detachment may form two types of seed banks, being (i) aerial (seeds attached to the maternal plant for an extended period of time after maturity) and (ii) soil seed bank (seeds at or below the soil surface) [1]. Usually, both types of seed bank play the same role, being to protect seeds from unfavorable conditions and releasing them once the conditions become favorable for germination and seedling establishment [2]. Each location is characterized by different exposures to predation, temperature extremes and salinity, thus increasing the temporal range of germination [1,3,4,5]. Aerial seed banks are common in species exposed to stresses of aridity [6,7], fire [8] and nutrient deficiency [9]. Aerial seed banks also occur in milder habitats [10], but they are more often studied in extreme conditions where they contribute to a species’ adaptation.

*Rumex obtusifolius* L (Polygonaceae) is one of the most widely distributed species in the world [11]. It colonizes modified habitats such as meadows, pastures, abandoned fields, roadside ditches, ruderal habitats and forest clearings up to 1500 m altitude [12]. It is a perennial herb growing to 40–150 cm and can produce large seed numbers that may remain viable in a soil seed bank for many years [13,14,15]. Mature seeds of *R. obtusifolius* may remain attached to the dry maternal plant, detaching in the following spring [16] to be dispersed by wind or animals [17]. Germination occurs in a wide range of environmental conditions [15,18], and the species can also reproduce vegetatively by root fragments or by underground stem [14,17,19]. In China, high infestation of *R. obtusifolius* in different habitats is negatively impacting agricultural productivity and is also suppressing native species diversity [20,21]. A better understanding of this species’ germination strategy could lead to more efficient weed control and habitat management programs.

The germination response of *R. obtusifolius* to light and temperature has been investigated previously [18,22]. A precise environmental characterization of temperatures, rainfall and wind speed are important for this work [23]. Other studies of seed burial depth on germination have found a significant negative correlation with seed germination [24,25,26,27]. In Japan, *R. obtusifolius* seeds remained viable under thick volcanic deposits for 10 years following the 1977–1978 eruptions of Mount Usu, Hokkaido Island [28]. Possible reasons for this longevity include that there were few predators, that seed removal by erosion or animal vector was rare, and that there was minimal competing vegetation. A later study 20 years after the eruption found a seed bank of at least 25 species with 2000 seeds per square meter, in which the dominant species was the non-native *R. obtusifolius* [29]. The study found a significantly positive correlation between *R. obtusifolius* seed viability and burial depth, suggesting that seed burial was protective over this time scale. From 20 to 30 years after the eruptions, species richness decreased based on the count model but increased according to the zero-hurdle model. The total seed number decreased over time, and *R. obtusifolius* seed density decreased, but seed frequency did not change [29]. Seedling emergence of *R. obtusifolius* requires burial at 8 cm or above, and seed dormancy is broken when the seed is exposed to temperatures above 20 °C for a few days [29]. More seeds were distributed in the upper layers of soil as shown in references in other studies [29,30,31]. Bhatt et al. [5] found that *Seidlitzia rosmarinus* and *Halothamnus iraqensis* seeds collected from a soil seed bank were 57% as viable as seeds collected from an aerial seed bank. For *H. iraqensis*, seeds in soil were 0% and in aerial seed banks, 100% viable. Traba et al. [32] describes that reduced germination of buried *Quercus ilex* seeds is depth-dependent. At 0–1 cm depth, seed germination was similar to non-buried seeds, but at 2–3 and 3–4 cm, germination was reduced by 90% and 95%, respectively. The influence of aerial vs. soil seed bank on germination has not been investigated in *R. obtusifolius*.

This study hypothesizes that seeds stored aerially might have a specific role that assists the species with an additional strategy to survive and proliferate even if the seeds diminish from the soil seed bank due to disturbance. Previous studies reported that R. *obtusifolius* seeds have the ability to remain viable in a soil seed bank for many years [13,28,33]. However, none of the studies evaluated the germination ecology of aerially retained seeds. Asynchronous seed release from aerial seed banks could be a mechanism to increase temporal variation in germination, thus ensuring some germination during optimal conditions. Generally, aerially retained seeds are exposed to greater variation of temperature and wind. Aerial seed banks facilitate temporal variation in seed dispersal but may also increase the uncertainty around the optimal time to germinate. Therefore, the objective of this study was to assess the germination responses of seeds stored in an aerial seed bank for different durations of temperature and light.

## 2. Results

**Figure 1:**
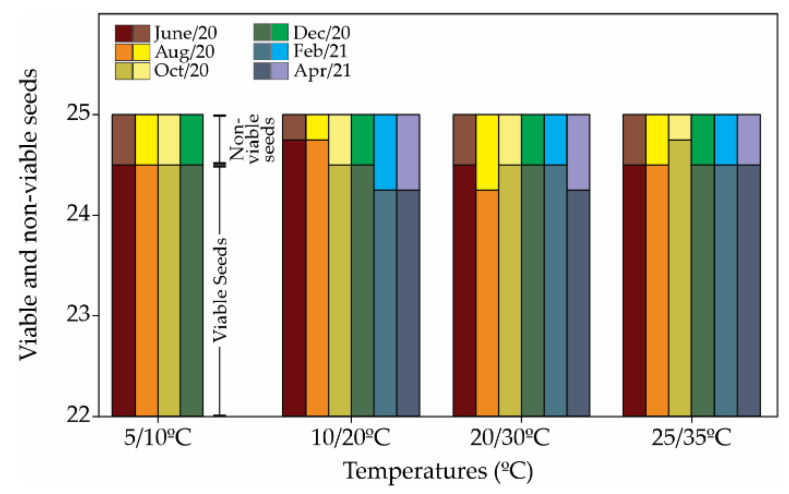
Viable and non-viable seeds computed after germination in 12 h light/12 h darkness in all temperatures. The non-viable seeds denote all seeds that were ungerminated after 30 days and were desiccated, as shown by dark embryos.

Morphological measurements included length, width, area, perimeter, weight, and the size and shape of *R. obtusifolius* seeds, which are tear-drop shaped, slightly flattened at the base (Supplementary Figure S1). Seed morphological parameters (length, width, height, and seed shape index) are summarized (Table 1). An average of 2.2% of seeds were non-viable (Figure 1) after the 30-day germination period at the end of the germination tests.

**Figure 2:**
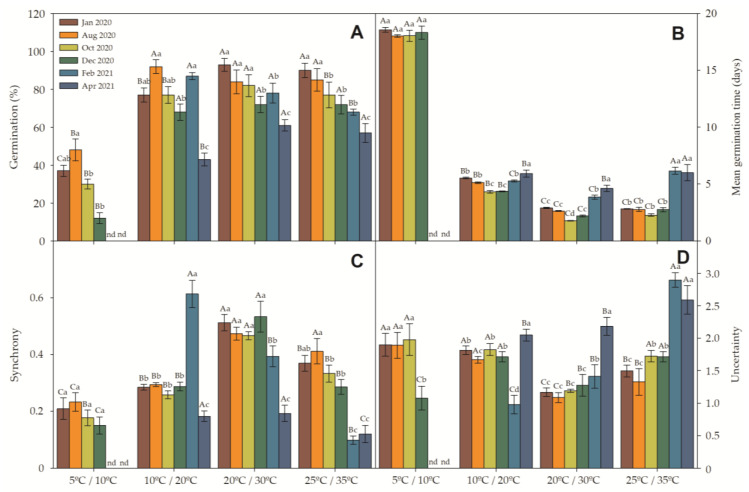
Seed germination (**A**) mean germination time (**B**) synchrony (**C**) and uncertainty (**D**) measured in *R. obtusifolius* seeds collected at six dates and incubated under four 12 h light/12 h darkness temperature regimes. Upper-case letters indicate differences (α = 0.01) among temperature regimes within collection dates, and lower-case letters indicate differences among collection dates within temperature regimes. All data denotes the mean (±SE). n = 4. nd = not determined.

All three factors (collection date, incubation temperature and incubation light exposure) and their interactions strongly affected germination rate (Table 2). Germination rate during 12 h light/12 h darkness declined with later collection dates (Figure 2A) under all temperature regimes (5/10 °C, *r* = 0.921, *p* = 0.003; 10/20 °C, *r* = 0.597, *p* = 0.008; 20/30 °C, *r* = 0.916, *p* = 5.4 × 10^−5^; 25/35 °C, *r* = 0.991, *p* = 3.1 × 10^−6^). Across all germination conditions, this represented a decline in germination from 74.3 ± 12.9 in fresh seeds to 40.3 ± 14.0% g in 10-month aerially stored seeds. The second collection date, August, had the highest overall germination rate due to better germination at lower temperatures (77.3 ± 9.9%). Germination was highest under the 20/30 °C temperature regime (78.3 ± 11.0%) and lowest under the coldest regime of 5/10 °C (21.2 ± 20.1%) (Figure 2).

In the seeds germinated in 24 h darkness, there was a weak significant correlation in the 10/20 °C (*r* = 0.606; *p* = 0.016; Table 3 and Table 4) and 20/30 °C (*r* = 0.577; *p* = 0.008) temperature regimes but not in the others. Excluding 5/10 °C data, which did not extend across all dates, germination rate was greatest on 8-month (73.0 ± 6.2%) and lowest on 10-month aerially stored seeds (36.7 ± 1.5). Cold (5/10 °C) germination conditions produced a low germination rate of 2.0 ± 3.6%, while germination at 10/20 °C was greatest in dark conditions, at 62.3 ± 8.2% (Table 3).

Mean germination time (MGT) was short (3–5 days) in all temperature regimes except the coldest, 5/10 °C, where it increased to 18.2 ± 0.26 days (Figure 2B). Regression of MGT with collection dates showed a significant relationship at 5/10 °C (*r* = 0.832; *p* = 0.012) and 10/20 °C (*r* = 0.200; *p* = 0.008) but not at 20/30 °C (*p* = 0.481) or 25/35 °C (*p* = 0.825). Higher temperatures lead to an increase in MGT, with all temperature regimes showing a similar regression, except for 5/10 °C. Reduced MGT would theoretically lead to greater germination synchrony, but seeds germinated at 5/10 °C showed no significant difference in SYN (Figure 2C). For seeds germinated at 10/20, 20/30 and 25/35 °C, there was a negative interaction between SYN and MGT, which persisted until the February, December and August collections, respectively.

Positive correlations (Table 4) were found between germination in light (12 h light/12 h darkness) and dark conditions (24 h darkness), MGT and UNC (Figure 2D), seed width and height, and seed weight, both in non-imbibed and 24 h-imbibed seeds. A negative correlation was found between SYN and both non-imbibed and 24 h-imbibed seeds. The decline in seed germination from the first to the last collection date was high at 5/10 °C (67.6%) and substantial at other temperatures: 44.2% (10/20 °C), 34.4% (20/30 °C) and 36.7% (25/35 °C).

**Figure 3:**
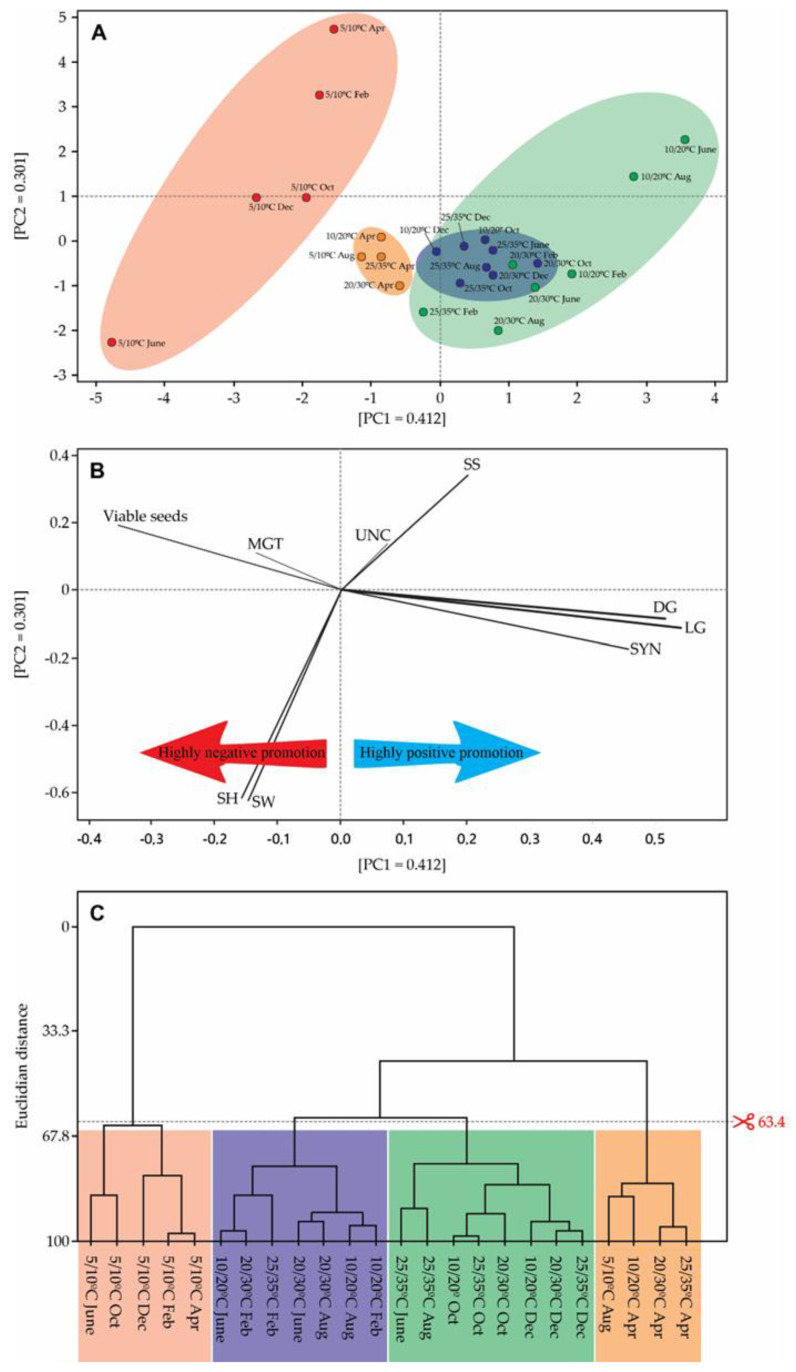
Multivariate analysis to assess the morphophysiological and germination parameters in *R. obtusifolius* comparing both 12 h light/12 h darkness and darkness (24 h darkness). (**A**) Treatments are displayed in the PC1 and PC2 to show that sample collection times there had a lesser influence on clustering than temperature regimes. (**B**) Spatial distribution of all analyzed features, showing the strength of each influencing the DG and LG. (**C**) Dendrogram showing how the clusters are formed and their similarities in strength. DG, germination in darkness; LG, germination in 12 h light/12 h darkness; SYN, Synchrony; SW, seed weight; SH, seed height; MGT, mean germination time; UNC, uncertainty; SS, seed shape.

All possible and significant correlations are shown in the multivariate analysis of the principal component analysis (PCA) (Figure 3). The analysis of component 1 (PC1) corresponds to the largest change in the parameter (41.2%), while component 2 (PC2) reflects the maximum parameter change in the same plane (30.1%), on which the location of the point with the coordinate of PC1/PC2 showed the state of seed germination. The PC1 and PC2 totally reflect 71.3% of changes (Figure 3A). In PC1, the most preponderant factors for explaining seed germination are SYN, which promotes germination, and SW and SH, which help to decrease the seed germination rate. However, in PC2, either SYN, SH, or SW promote the germination, while in viable seeds, MGT, UNC and SS corroborate to decrease seed germination (Figure 3B). The strength with which each of the factors influence both germination in light and dark are highlighted with different strengths in Figure 3B (thicker and softer lines). Supplementary Table S1 shows the strength that each of the factors analyzed weigh in the promotion (positive values) or delay/inhibition (negative values) of the germination of the species. Figure 3C shows all clusters formed after PCA analysis showing the formation of 4 groups, with sharing with group 2 and 3 (Figure 3C).

## 3. Materials and Methods

### 3.1. Collection

Mature fruits of *Rumex obtusifolius* were collected from a field at Guling, Jianxi, China (29°32′55″ N, 115°58′46″ E, 1057 m a.s.l.) in June 2020 (time 0) and after 2 (August 2020), 4 (October 2020), 6 (December 2020), 8 (February 2021), and 10 (April 2021) months of aerial storage. Collection ceased after April 2021 since the aerial seed bank was mostly depleted. Seeds were collected from 15 to 20 maternal plants spaced at least 2 m apart to ensure representation of genetic diversity as described previously [34]. Perianths were removed from fruits at collection time, and seeds were tested for germination within a week after each collection. Temperature and rainfall data were obtained from the Jiujiang Meteorological Bureau, Jiangxi, China.

### 3.2. Morphology

Seed shape index and seed dimensions (length, width, height) were assessed on 15 seeds per collection date using a Stereo Microscope (Nikon SMZ800N; Nikon Instruments Inc. Melville, NY, USA) coupled with a microscope camera IMG-SC600C (iMG Biotechnology Co., Ltd., Suzhou, Jiangsu, China). For this, seeds were attached ventrally to filter-paper using double-sided sticky tape. Seed mass was determined at time of collection from three 1000-seed replicates, using an analytical balance (Sartorius Analytical Balance mod. ENTRIS224-1S, Bradford, MA, USA; accurate to 0.1 mg). Seed shape index was calculated as described in Thompson, et al. [35]. In this methodology, the values may range from 0 (in perfectly spherical seeds) to a maximum value of about 0.3 (in needle- or disc-shaped seeds).

### 3.3. Water Imbibition

Seed permeability to water was assessed on three 25-seed replicates by observing mass before and after a 24 h incubation at 25 °C with 15 mL deionized water. Water absorption was expressed as a percentage change in mass [36]. Previous experimentation had determined that seed fresh mass did not change further after 24 h exposure.

### 3.4. Temperature and Light

**Figure 4:**
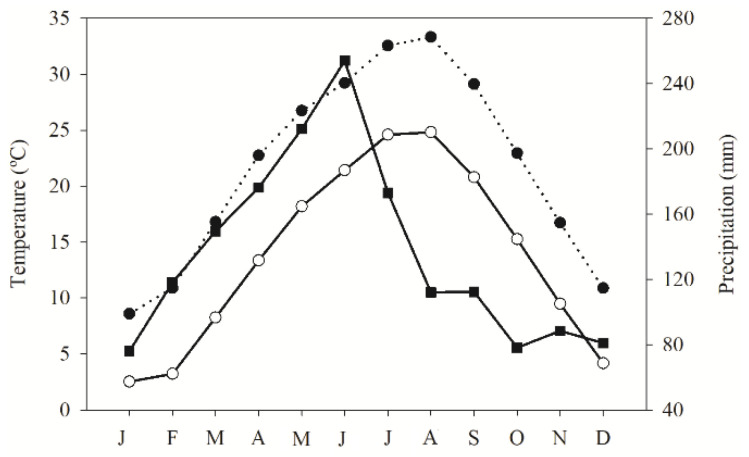
Minimum temperature (white circles with bold line), maximum temperature (black circles with dotted line) and precipitation (black squares with bold line), registered as mean of the five years after the start of experiment (2015 to 2019) for Jiujiang, Jiangxi, China. Source: Jiujiang Meteorological Bureau, Jiangxi, China.

Seed surfaces were sterilized in 0.50% sodium hypochlorite for 1 min, then washed thrice with deionized water to avoid fungus infection. Seeds from each of the six collection dates were germinated at four temperature regimes (12/12 h of 5/10, 10/20, 20/30 and 25/35 °C) and two light regimes (either 12 h light/12 h darkness (light treatment) or 24 h darkness (dark treatment)). Germination tests were performed in Kesheng incubators (Model-DRX-800C-LED, China) fitted with cool-white, fluorescent tubes (60 µmol photons m^−2^ s^−1^). Temperature regimes were selected to simulate outdoor night/day conditions throughout the year (Figure 4), representing December to February (5/10 °C), March to April and October to November (10/20 °C), May to June and September (20/30 °C), and July to August (25/35 °C). The lowest temperature regime (5/10 °C) was discontinued for the final two collection dates due to low seed reserves in the aerial bank and low percentage of germination in the first four.

Seeds were placed in 9 cm Petri dishes containing one disk of Whatman No. 1 filter paper moistened with 10 mL of distilled water. Darkness was simulated by wrapping Petri dishes in two layers of aluminum foil. Four replicates of 25 seeds each were used for each treatment. Germination was defined as the protrusion of a radicle by ≥2 mm through the external integument [37]. Seed germination was observed daily in the light treatment (12 h light/12 h darkness) for 30 days after seed soaking, but in the dark treatment was observed at day 30. Germination percentages, mean germination time (MGT), synchrony (SYN) and uncertainty (UNC) were computed using GerminaQuant R package [38]. As the germination in darkness does not allow viewing the seeds that germinated on each *i*th day, the MGT, the synchrony and the uncertainty could only be calculated in the seeds that were germinated in the light (12 h light/12 h darkness). This differential data collection between treatments is due to the requirement that seeds in the dark treatment not be exposed to any light during the germination period [5,38,39,40]. At the end of the germination test, ungerminated seeds from the light treatment (12 h light/12 h darkness) were dissected and examined under a stereoscope for embryo viability, where white indicates viable and brown indicates dead.

### 3.5. Data Analysis

The influence of collection date, incubation temperature and incubation light conditions on four dependent variables (germination rate, mean germination time, synchrony, and uncertainty) were assessed by ANOVA. Synchrony $=\frac{\sum C_{n_{i},2}}{N}$, being $c_{n_{i},2}=\frac{n_{i}\left(n_{i}-1\right)}{2}$ and $N=\frac{\sum n_{i}(\sum n_{i}-1)}{2}$; $\mathrm{Uncertainity}=-\sum_{i=1}^{k}f_{i}\log_{2}f_{i}$, being $f_{i}=\frac{n_{i}}{\sum_{i=1}^{k}n_{i}}$. Where, *n_i_* is the number of seeds germinated on the ith time, *t_i_* is the number of days from beginning of the germination test to the ith observation, *X_i_* is the period of germination experiments, *k* is the final day that germination was scored, *f_i_* is the relative germination frequency, and $C_{n_{i},2}$ is the number of seeds germinated on the *i*th time interval.

Correlations among continuous variables were assessed using Pearson correlations. Principal component analysis (PCA) was performed using Pearson’s correlation and Ward’s method to identify the variable(s) that best explained the highest proportion of total variance using Minitab^®^ version 17.0 software (Minitab Ltd. Coventry, UK). Analyses were performed using SPSS 26 (IBM Corporation, Armonk, NY, USA).

## 4. Discussion

Temperature and moisture are the main factors determining seed viability during storage [39]. However, both temperature and precipitation during winter and summer did not reduce the seed viability. There was also a strong correlation between the months of collection and germination, both in 12 h light/12 h darkness and 24 h darkness. However, we believe that if the process of germination occurred in the natural environment at the same temperature at which the germination of *R. obtusifolius* seeds was simulated in the laboratory, the reduction in germination rate would be even more pronounced, as the laboratory conditions may not fully replicate the complex and dynamic interactions that occur in the natural environment. In nature, seeds are exposed to various factors like fluctuations in temperature, humidity, light and interactions with other organisms. These factors can have both positive and negative effects on germination [41].

Seeds exhibited the greatest germination rates when harvested from the aerial seed bank soon after maturity but before dispersal (June 2020). Those remaining in the aerial seed bank for 10 months before harvesting (April 2021) had the lowest germination rates. Seeds remaining in the seed bank after June 2020 were probably subjected to a lower water potential because they were exposed to lower temperatures over time. It is likely that respiration rate was low, preventing seed vigor decline over time. Respiration rate is reduced by low temperatures [40], which partially explains seed viability after 12 months of aerial storage. Seeds remaining on the aerial seed bank might induce the secondary dormancy. It is a mechanism that inhibits germination even when conditions for germination are favorable. It acts as a protective strategy for seeds to ensure their survival in unfavorable or uncertain environmental conditions [42]. However, during storage, the seeds may experience hydration–dehydration cycles as well as extreme temperature fluctuation (21.4 °C in June 2020 to 2.5 °C in February 2021), which may have a priming effect on seed physiology resulting in enhanced germinability [43,44,45]. Previous studies reported that *R. obtusifolius* seeds germinate throughout the year, though seedling emergence peaks in spring [46]. This season-sensing mechanism would enhance seedling emergence and survival by avoiding germination during unfavorable seasons. Soon after germination, ADP may contribute to the production of adenosine triphosphate (ATP) [47]. This metabolic pathway is dependent on water and enzyme activity and thus depletes the seed reserves, resulting in lower seed germination. Thus, cold months, despite high humidity, may have contributed to a lower metabolic rate of hydrolytic enzymes, preserving the reserves for future germination, since the germination of *R. obtusifolius* seeds had a moderate average reduction of 36.7 ± 7.6% after 10 months of storage in the seed banks. Different results were described by Moncaleano-Escandon, et al. [48] who described an almost 100% decline in the germination rate of *Jatropha curcas* seeds after 12 months, without the use of desiccants. However, when the seed storage was combined with a desiccant (silica gel), the maintenance of germinability after 12 months was similar to fresh seeds (reduction of 33%, non-significant) [49]. These authors postulate that the maintenance of seed germination without a significant fall was due to a decrease in the osmotic potential of −35 MPa (fresh seeds) to −125 MPa in 12-month seeds. The reduction of osmosis provoked a reduction in the respiratory rate from 114 to 10 mmol CO_2_ g^−1^ h^−1^. Similar behavior was described for *Sorghum bicolor* seeds [50], where the authors showed a high regression coefficient (R^2^) between the respiratory rate (44 μmol CO_2_ g^−1^ h^−1^) and the germination rate (85%).

In the present study, freshly harvested *R. obtusifolius* seeds germinated to 92% with a low MGT of two to five days, indicating that *R. obtusifolius* seeds are non-dormant. However, previous studies reported that freshly mature seeds of *R. obtusifolius* have a high degree of dormancy [14,26]. These discrepancies could be due to the variation in local environmental conditions or genetic differences [51,52,53]. Overall, seeds of *R. obtusifolius* were able to germinate well except at the lowest temperature (5/10 °C). This ability to germinate well over a wide range of simulated seasonal temperatures is expected because this species is able to persist well in tropical, subtropical and temperate biomes. However, the lower germination percentage at lower temperatures indicates avoidance of germination during winter, when low temperatures reduce seed metabolic rates [44] and prevent hydrolysis of reserves, necessary for germination. Seedling mortality in winter is likely to be higher due to frosts. In the present study, seeds were collected from 1000 m a.s.l., where winter temperature is very low and chances of snowfall are high.

Aerially retained seeds remained highly viable throughout the collection period, indicating tolerance to seasonal weather changes. This suggests a strong resilience of *R. obtusifolius* in many parts of the world that makes it a difficult weed to control. However, seeds collected at different times from the aerial seed bank showed significant variation in germination, with the highest percentage in August, after a 2-month storage.

Uncertainty was proposed by Shannon [54] to measure the informational entropy or uncertainty associated with the frequency distribution. This expression is used by ecologists to measure the diversity of one environment [39]. Thus, high values for this index indicate high diversity. When applied to seed germination, the conventional interpretation is in the opposite direction. That is, low values indicate more synchronized germination. Low values of UNC indicate frequencies with few peaks, that is, germination more concentrated in time. UNC measures the degree of spreading of germination through time and can be used, by inference, to measure the synchrony of germination. On the contrary, SYN produces a number if only there are two seeds finishing the germination process at the same time. Thus, the value of SYN measures the synchrony of germination [39]. Low values of MGT and high synchrony, combined with low uncertainty, confirm our hypothesis that *R. obtusifolius* seeds are very resistant to cold and capable of germinating over time with high rates.

Principal Component Analysis (PCA) is a powerful tool that enables isolation of a single factor (in this specific case, 12 h light/12 h darkness and 24 h darkness) and analyzes which other factors directly or inversely influence its shape (12 h light/12 h darkness and 24 h darkness) [55]. In addition to demonstrating the strength with which each component participates in the promotion or delay/inhibition of a specific factor (in the present case, LG and DG), the PCA allows us to divide the applied treatments by their similarity with another treatment [56]. In the present work, SYN and SS appeared to be the main factors promoting germination, while UNC contributed to a lesser extent. For invasive species such as *R. obtusifolius*, having greater synchronization in germination can be favorable, as it takes advantage of a certain moment in the biogeochemical cycle of the environment, as well as favoring seed dispersal within favorable conditions for the species. Acting as a form of biological control, high synchrony is desired, while in more anthropized environments or with a certain manipulation by people, favoring the entropy of the environment, the species adapted to this environment can launch its seeds in a short time, to germinate in a short period of time if that is the favorable condition or else to disperse the seeds in longer periods to prevent a large batch of seeds from being lost due to the simple fact that they were dispersed all at the same time, when conditions are not favorable for germination. On the other hand, MGT, acting with SYN, favors longer or shorter germination depending on the environmental conditions. Normally, these two variables are antagonistic because a higher germination rate normally leads to a faster and more synchronous germination [57]. It is important to emphasize that the highest MGT is an excellent tool to assess the speed of germination, even if it takes longer to happen (higher MGT) or presents a faster germination to take advantage of the natural conditions of the environment. In the case of *R. obtusifolius*, Figure 2 clearly shows its antagonistic effect. When it comes to PC2, the most important factors favoring the germination of *R. obtusifolius* are SYN, SH and SW. SH and SW clearly show us that slightly heavier seeds have a favored faster germination, while lighter seeds may present slower germination, a fact that was recently demonstrated in a study with nine other invasive species from China [58]. *Arthrocnemum macrostachyum* and *Suaeda vermiculata*, other invasive species from China, showed the same behavior [34] as well as 19 other species studied [59] in the same climate and natural conditions as those presented in this work. However, this antagonistic phenomenon is not restricted to species of arid climate.

Lara, et al. [60], studying the effect of the source of nitrate used on the germination of *Lycopersicon esculentum*, also showed a higher MGT with lower SYN values. Eight different pasture-forming grasses also showed the same antagonistic behavior between SYN and TMG [58]. In this same PCA, we can infer that larger seeds have a lower germination with a lower synchrony. In this item, the current bibliography does not allow us to make a direct inference, since larger or smaller seeds can present different germination strategies, with larger seeds presenting larger or smaller SYN [34,58].

## 5. Conclusions

Formation of a transient aerial seed bank increases long-term fitness and reduces risk of germination failure by avoiding germination synchrony. Seeds were able to remain highly viable throughout the collection period, although germination ability varied among collection times. This indicates that seed viability remains unaffected by seasonally fluctuating weather conditions. The strong resilience of *R. obtusifolius* to germinate in many parts of the world is an obstacle for effective weed control.

## Figures and Tables

**Table 1:** *R. obtusifolius* seed morphology. Imbibed seed weight was determined after soaking seeds for 24 h.

Feature	Mean (±SE)
Non-imbibed seed weight (g)	0.027 ± 0.003
24 h-imbibed seed weight (g)	0.031 ± 0.002
Seed length (mm)	2.193 ± 0.033
Seed width (mm)	1.351 ± 0.020
Seed height (mm)	1.343 ± 0.018
Seed shape index	0.033 ± 0.002
1000-seeds weight (g)	1.090 ± 0.104

**Table 2:** Analysis of variance of collection date, germination temperature and germination light exposure on seed germination of *R. obtusifolius*.

Source of Variation	Degrees of Freedom Residuals	Sum of Squares	Mean Squares	Significance (*p*)
Date	5	19,091.42	3818.28	<0.001
Temperature	3	110,324.92	36,774.97	<0.001
Light	1	18,174.08	18,174.08	<0.001
Date × Temperature	15	6929.58	461,97	<0.001
Date × Light	5	3745.42	749.08	<0.001
Temperature × Light	3	1742.92	580.97	<0.001
Date × Temperature × Light	15	4667.58	311.17	<0.001
Residuals	144	3051.50	21.19	---

**Table 3:** Seed germination of *R. obtusifolius* comparing the 12 h light/12 h darkness and darkness (24 h darkness) treatment in each temperature of incubation and sample data. All values denote median (±SE). n = 4. Values followed by ns denote non-significant differences in germination between light (12 h light/12 h darkness) and darkness (24 h darkness), * significant at *p* < 0.05, ** significant at *p* < 0.01, and *** significant at *p* < 0.001, verified by *t*-test. nd denote not determined.

Temperature	12 h Light/12 h Darkness						Full Darkness (0 h)					
(°C)	June/20	Aug/20	Oct/20	Dec/20	Feb/21	Apr/21	June/20	Aug/20	Oct/20	Dec/20	Feb/21	Apr/21
5 °C/10 °C	37 ± 3.0 ***	48.0 ± 11.8 **	30.0 ± 2.6 ***	12.0 ± 2.8 ***	nd	nd	1.0 ± 1.0	7.0 ± 1.9	0	0	nd	nd
10 °C/20 °C	77.0 ± 3.8 ^nd^	92.0 ± 3.7 ^ns^	77.0 ± 4.4 **	68.0 ± 4.3 **	87.0 ± 1.9 *	43.0 ± 3.4 ^ns^	77.0 ± 2.5	85.0 ± 3.0	52.0 ± 3.3	45.0 ± 2.5	78.0 ± 2.6	37.0 ± 3.4
20 °C/30 °C	93.0 ± 3.4 ***	84.0 ± 6.3 ^ns^	82.0 ± 5.8 *	72.0 ± 4.3 **	78.0 ± 5.3 ^ns^	61.0 ± 3.0 **	70.0 ± 2.6	68.0 ± 5.2	61.0 ± 1.9	51.0 ± 3.4	75.0 ± 3.4	38.0 ± 4.8
25 °C/35 °C	90.0 ± 3.8 ***	85.0 ± 6.0 ***	77.0 ± 6.8 *	72.0 ± 4.9 **	68.0 ± 1.6 ^ns^	57.0 ± 5.0 **	33.0 ± 1.9	44.0 ± 3.4	53.0 ± 6.6	47.0 ± 3.4	66.0 ± 2.6	35.0 ± 1.9

**Table 4:** Pearson correlations (*r*) of traits measured on *R. obtusifolius* collected in June, August, October and December 2020 and February and April 2021 for temperature regimes (5 °C/10 °C; 10 °C/20 °C; 20 °C/30 °C, and 25 °C/35 °C). Significance is *p* ≤ 0.5 (*) and *p* ≤ 0.1 (**) and after application of the Bonferroni correction occurs at (α = 9.1 × 10^−4^), the former by asterisk and the latter by shading, ns = not significant.

Germination in light											
Germination in darkness	0.83 **										
Mean germination time (light)	−0.2 ^ns^	−0.3 ^ns^									
Uncertainty (light)	−0.2 *	−0.2 ^ns^	0.36 **								
Synchrony (light)	0.68 *	0.37 ^ns^	−0.1 ^ns^	−0.3 ^ns^							
Seed length	0.34 ^ns^	0.09 ^ns^	−0.2 ^ns^	0.01 ^ns^	0.11 ^ns^						
Seed width	−0.2 ^ns^	−0.7 ^ns^	0.14 ^ns^	0.13 ^ns^	0.19 ^ns^	0.03 ^ns^					
Seed height	0.56 ^ns^	−0.1 ^ns^	0.12 ^ns^	0.08 ^ns^	−0.1 ^ns^	0.16 ^ns^	0.95 **				
Seed weight (0 h)	0.79 ^ns^	−0.1 ^ns^	0.03 ^ns^	0.9 ^ns^	−0.9 **	−0.4 ^ns^	0.61 ^ns^	0.51 ^ns^			
Seed weight (24 h)	0.79 ^ns^	0.19 ^ns^	0.33 ^ns^	0.99 ^ns^	0.7 *	0.26 ^ns^	0.82 ^ns^	0.75 ^ns^	0.95 ^ns^		
1000-seed weight	0.535 ^ns^	0.562 ^ns^	0.03 ^ns^	0.9 ^ns^	−0.3 ^ns^	−0.3 ^ns^	0.61 ^ns^	0.51 ^ns^	0.998 **	0.95 ^ns^	
	Germination in light	Germination in darkness	Mean germination time (light)	Uncertainty (light)	Synchrony (light)	Seed length	Seed width	Seed height	Seed weight (0 h)	Seed weight (24 h)	1000-seed weight

## Data Availability

Not applicable.

## Supplementary Materials

The following supporting information can be downloaded at: https://www.mdpi.com/article/10.3390/plants12132403/s1, Supplementary Figure S1: *Rumex obtusifolius* seed size. Supplementary Table S1. All ranks that promote or inhibit/delay the seed germination, both in 12-h light/12-h darkness and darkness (24-h darkness). Higher and lower values promote or inhibit/delay seed germination. PC1 is strongest component, followed by PC2.
