# Supplementary material for: Storage on Maternal Plants Affects Temperature Requirements during Germination in Rumex obtusifolius
Source: Plants (Basel). 2023 Jun 21;12(13):2403. doi: 10.3390/plants12132403 (PMC10346210; doi:10.3390/plants12132403)
Supplement: Supplementary file 1 [file plants-12-02403-s001.zip › plants-2384801-supplementary.pdf]

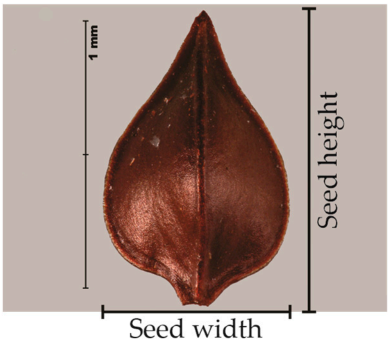

Supplementary Figure S1. *Rumex obtusifolius* seed size.

Supplementary Table S1. All ranks that promote or inhibit/delay the seed germination, both in 12-h light/12-h darkness and darkness (24-h darkness). Higher and lower values promote or inhibit/delay seed germination. PC1 is strongest component, followed by PC2.

Variable	PC1	PC2	PC3	PC4	PC5	PC6	PC7	PC8	PC9
LG	0.463	-0.281	0.152	-0.186	0.039	-0.291	0.142	-0.729	-0.106
DG	0.488	-0.130	0.056	-0.248	0.130	0.339	-0.665	0.134	0.296
TMG	-0.126	-0.475	-0.255	0.307	0.757	0.141	0.074	-0.028	-0.009
UNC	0.059	-0.501	-0.353	-0.493	-0.156	-0.349	0.146	0.453	-0.071
SYN	0.382	-0.279	0.333	0.221	-0.220	0.496	0.467	0.270	-0.175
SW	-0.292	-0.313	0.514	0.057	-0.026	-0.155	-0.449	0.083	-0.563
SH	-0.333	-0.361	0.434	0.021	-0.125	-0.111	0.090	-0.038	0.730
Viable Seed	-0.290	-0.330	-0.424	0.048	-0.496	0.440	-0.180	-0.387	-0.060
SS	0.319	-0.115	-0.203	0.715	-0.270	-0.427	-0.224	0.119	0.111
